# Beyond Positive or Negative: Latent Profiles of University Students’ AI Attitude and Their Career-Development Correlates

**DOI:** 10.3390/bs16091460

**Published:** 2026-08-22

**Authors:** Hongfeng Song, Anqi Hu, Xueyan Li

**Affiliations:** 1 School of Economics and Management, Beijing Forestry University, Beijing 100083, China; songhf@bjfu.edu.cn; 2 School of Labor and Human Resources, Renmin University of China, No. 59 Zhongguancun Avenue, Haidian District, Beijing 100872, China; anqihu@ruc.edu.cn

**Keywords:** artificial intelligence, AI attitude, latent profile analysis, career crafting, perceived employability

## Abstract

Artificial intelligence (AI) can be appraised simultaneously as useful, capable, threatening, and demanding. Yet research typically treats attitudes toward AI as a single favorable-unfavorable continuum, obscuring how these evaluations coexist within individuals. Using a three-wave, time-lagged survey of 379 first-year university students in China, we examined configurations of six AI-related appraisal indicators: perceived humanlikeness, adaptability, and quality of AI; AI use anxiety; awareness of smart technology, artificial intelligence, robotics, and algorithms (STARA) as a career threat; and AI creative self-efficacy. Latent profile analysis supported four profiles: Positive Empowerment (19.2%), Anxious Acceptance (37.5%), Low-Perception Detached (37.2%), and High-Perception High-Vigilance (6.1%). R3STEP models showed that learning agility, digital literacy, future work self salience, and perceived university digital support were prospectively associated with profile membership, particularly in distinguishing the Low-Perception Detached profile from the more engaged profiles. BCH comparisons further showed that the Positive Empowerment and High-Perception High-Vigilance profiles reported relatively high levels of both career crafting and self-perceived employability, whereas the Low-Perception Detached profile reported the lowest levels of both outcomes. Notably, the High-Perception High-Vigilance profile combined elevated AI use anxiety and STARA awareness with strong career-development engagement, while the Low-Perception Detached profile combined comparatively low threat perceptions with weak career crafting and employability. These findings demonstrate that higher AI-related threat was not necessarily associated with weaker career preparation and, conversely, that low perceived threat was not necessarily associated with greater adaptive readiness. Overall, the results position students’ responses to AI as configurations of opportunity appraisal, threat, and AI creative self-efficacy rather than as uniformly positive or negative attitudes, thereby highlighting the need for differentiated university career education and AI-readiness interventions.

## 1. Introduction

Artificial intelligence is changing how people learn, search for work, make decisions, and imagine future occupations. For university students who are beginning to construct vocational identities, AI is therefore more than a new tool. It is also a prospective collaborator, a source of uncertainty, and a signal about which capabilities may remain valuable in the labor market. Existing research has shown that people differ in their willingness to rely on algorithms, the extent to which they attribute humanlike qualities to AI, and the degree to which they anticipate technological displacement (Brougham & Haar, 2018; Dietvorst et al., 2015; Glikson & Woolley, 2020; Park & Woo, 2022). These reactions matter because attitudes guide attention, behavioral intentions, and engagement with an attitude object (Eagly & Chaiken, 1993; Park et al., 2024). For students, the same reactions may shape whether they actively prepare for AI-affected careers or withdraw from that preparation.

Research on university students’ AI attitudes has largely adopted a variable-centered approach, emphasizing discrete acceptance-related factors such as perceived usefulness, ease of use, trust, anxiety, and behavioral intention (Chan & Hu, 2023; Budhathoki et al., 2024; Maheshwari, 2024; Strzelecki, 2024). Related research has examined anthropomorphic inferences about nonhuman agents (Epley et al., 2007), algorithm aversion following observed errors (Dietvorst et al., 2015), awareness of the occupational consequences of STARA (Brougham & Haar, 2018), and trust in AI systems (Glikson & Woolley, 2020). Collectively, these studies show that AI attitudes encompass instrumental evaluations, social perceptions, affective reactions, and career-related concerns. However, these dimensions are typically examined separately or summarized through average associations, offering limited insight into how they combine within individuals.

This limitation is theoretically consequential because favorable and unfavorable reactions to AI are not necessarily opposite ends of a single continuum. Existing measures distinguish positive evaluations from negative concerns and differentiate capability appraisals, AI use anxiety, job insecurity, and personal utility as related but distinct components of AI attitudes (Schepman & Rodway, 2020; Park et al., 2024). Students may therefore view AI as capable and useful while also experiencing anxiety or anticipating career disruption. Variable-centered analyses can estimate average associations among these dimensions, but they cannot identify the subgroups in which such reactions co-occur. Zhang et al. (2025) provided initial person-centered evidence by identifying distinct profiles of university students’ attitudes toward ChatGPT and linking them to use intentions. The present study extends this work by examining how multiple components within a broader AI-attitudinal system combine within individuals, including perceptions of AI capability, use-related anxiety and career-related threat, and AI creative self-efficacy. It also shifts the focus from intentions to use a specific AI tool to the career-development relevance of AI attitude profiles, as reflected in career crafting and self-perceived employability.

Building on this multidimensional and person-centered premise, Park et al.’s (2024) Attitudes Towards Artificial Intelligence at Work (AAAW) framework provides the primary conceptual foundation for the present study. Because the framework was developed for workplace settings, we adapt its multidimensional logic to university students preparing for work. The selected indicators capture three broad domains: evaluations of AI capability, perceived use-related and career-related threat, and AI creative self-efficacy.

We make three contributions. First, we shift the study of AI attitudes from a positive-negative continuum to person-level configurations of cognitive appraisal, affective threat, and efficacy. Second, we connect these configurations to vocational development by examining whether learning agility, digital literacy, future work self salience, and perceived university digital support differentiate profile membership. Third, we examine whether the profiles are associated with career crafting and self-perceived employability. In doing so, the study links an emerging AI attitudes literature to core questions in vocational behavior concerning proactive career preparation, anticipated labor-market threat, and confidence in obtaining future work.

## 2. Theoretical Framework

### 2.1. AI as an Opportunity, a Threat, or Both?

Attitude is a psychological tendency expressed through the evaluation of a particular entity with some degree of favor or disfavor; such evaluations may be reflected in cognitive and affective responses (Eagly & Chaiken, 1993, 2007). AI is a particularly complex attitude object because it can be understood simultaneously as a technological tool, a decision aid, a quasi-social actor, and a force reshaping education and work.

The coexistence of favorable and unfavorable reactions is consistent with broader attitude and appraisal theories. The evaluative-space model proposes that positive and negative evaluations can be activated partly independently rather than functioning as mutually exclusive endpoints (Cacioppo & Berntson, 1994). Cognitive appraisal theory similarly suggests that the same environmental change may be evaluated as both a potential gain and a potential threat, depending on its perceived significance and the resources available to manage it (Lazarus & Folkman, 1984). In the case of AI, capabilities that enable augmentation, efficiency, and creativity may also indicate automation, substitution, or reduced human control—the central tension captured by the automation-augmentation paradox (Raisch & Krakowski, 2021). In the present study, the positive side of this bivalent orientation is reflected most directly in AI creative self-efficacy and is complemented by stronger perceptions of AI capability, particularly adaptability and quality, whereas AI use anxiety and STARA awareness capture negative affective and career-related threat responses. Perceived humanlikeness is treated as a capability-related perception rather than as inherently positive.

University students preparing to enter the labor market may regard AI as highly capable and useful while also worrying about keeping pace with technological change or about the future value of human skills. Conversely, low anxiety or low perceived career threat may reflect confidence and preparedness, but it may also reflect limited exposure, weak awareness, or disengagement. The theoretically important question is therefore not simply whether students hold positive or negative attitudes toward AI, but how evaluations of AI capability, perceived threat, and AI creative self-efficacy are combined.

### 2.2. A Multidimensional Framework of AI Attitudes

Park et al.’s (2024) AAAW framework conceptualizes attitudes toward AI as a multidimensional system rather than a single favorable-unfavorable evaluation. Drawing on Eagly and Chaiken’s (1993, 2007) definition of attitude, Park et al. (2024) note that evaluation of an attitude object encompasses “cognitive and affective components such as beliefs, thoughts, feelings and emotions”. Importantly, their goal was not to treat the six AAAW dimensions as interchangeable manifestations of one latent factor, but to capture “distinct and related attitudinal constructs under the broad theme of AAAW”.

Park et al.’s (2024) original framework comprises six dimensions: perceived humanlikeness, perceived adaptability, perceived quality, AI use anxiety, job insecurity, and personal utility. Guided by this multidimensional structure, the present study draws on the first four indicators in student-contextualized form, while using STARA awareness to represent career-related technological threat and AI creative self-efficacy to capture students’ beliefs about their capability to use AI creatively.

A defining feature of this broader attitudinal system is that all six indicators are explicitly anchored to the same attitude object—AI and individuals’ interaction with AI. Although they differ in psychological content, they represent distinct cognitive, affective, threat-related, and self-referent responses to AI. Thus, the dimensions capture different object-specific components that may combine in distinct ways within individuals.

*Perceptions of AI Capability.* Perceived humanlikeness, adaptability, and quality capture students’ evaluations of what AI is and what it can do. Humanlikeness concerns the attribution of humanlike communicative or social qualities to AI, whereas adaptability and quality refer to its perceived responsiveness, reliability, and effectiveness across tasks (Epley et al., 2007; Glikson & Woolley, 2020; Park et al., 2024). Park et al. (2024) link heightened humanlikeness to perceptions that AI can observe, think, and evaluate more effectively, while treating adaptability and quality as functionality-related aspects of AI. Thus, higher scores indicate stronger perceptions of AI capability, although these perceptions are not assumed to be uniformly positively valenced. In particular, capability may be appraised as useful and enabling while simultaneously signaling greater potential for automation and substitution (Raisch & Krakowski, 2021).

*Use-Related Anxiety and Career-Related Threat.* AI use anxiety and STARA awareness capture distinct forms of perceived threat. AI use anxiety reflects tension associated with learning and operating AI technologies, whereas STARA awareness concerns the broader occupational implications of smart technologies, artificial intelligence, robotics, and algorithms (Brougham & Haar, 2018; Wang & Wang, 2022). Students may feel comfortable using AI while remaining alert to its career consequences, or experience use-related anxiety without fully recognizing its labor-market implications. Moreover, elevated threat may indicate either vulnerability or informed vigilance, depending on the personal resources with which it co-occurs.

*AI Creative Self-Efficacy.* The original AAAW framework includes personal utility, defined as “a perception of usefulness and satisfaction of using AI at work based on perceived value and expectation” (Park et al., 2024). This dimension contains a clear self-referent component, encompassing the extent to which AI use is expected to support workers’ “confidence, independence and feelings of enjoyment at work”, as well as feelings of accomplishment and control. Because these items were developed for incumbent workers and are framed around experiences within an established work role, directly transferring them to first-year university students would require students to evaluate forms of workplace utility that they may not yet have personally experienced.

In the present study, we therefore incorporated AI creative self-efficacy as a student-contextualized indicator of this self-referent aspect of AI engagement. Whereas personal utility concerns the perceived value and expected benefits that AI use may provide to the individual, creative self-efficacy refers to individuals’ beliefs that they can be creative in their work roles (Bandura, 1997; Tierney & Farmer, 2002), which we contextualized as students’ confidence in their capability to use AI creatively in academic work. Thus, both constructs involve self-referent expectations concerning engagement with the focal technology—what AI use may enable for the individual and what the individual believes they can accomplish through AI use. We therefore do not regard AI creative self-efficacy as equivalent to personal utility; rather, it represents a theoretically aligned, contextually adapted indicator that preserves the original framework’s emphasis on the personally relevant implications of AI use. Although AI creative self-efficacy is a self-referent belief rather than a direct evaluation of AI, higher levels represent a clearly positive efficacy-based orientation toward AI use: students believe that engaging with AI can enable them to produce more creative academic outcomes.

A person-centered approach can identify subgroups characterized by similar patterns across multiple indicators. Latent profile analysis (LPA) can distinguish level differences, in which profiles vary mainly in their overall elevation, from shape differences, in which particular dimensions are relatively high or low within a profile (Morin & Marsh, 2015; Spurk et al., 2020). This distinction is central to the present study because students with comparable overall levels of favorable AI attitudes may differ substantially in anxiety, career-related threat, or AI creative self-efficacy.

Several configurations are theoretically plausible. An opportunity-dominant configuration may combine favorable capability evaluations and high AI creative self-efficacy with relatively low threat. An opportunity and threat coactivation configuration may combine favorable evaluations and high AI creative self-efficacy with elevated anxiety or career vigilance, reflecting simultaneous recognition of AI’s benefits and disruptive potential. A threat-dominant configuration may involve elevated threat alongside low AI creative self-efficacy, suggesting that students perceive technological change as difficult to translate into personally valuable action. Finally, a detached configuration may combine low capability evaluations, low threat, and low AI creative self-efficacy, indicating limited engagement rather than genuine preparedness. More moderate or differently shaped configurations may also emerge.

These configurations are not proposed as predetermined hypotheses. Rather, they represent theoretically informed examples of how AI attitudes may combine among university students. They provide a conceptual basis for anticipating and interpreting possible profile patterns without imposing a fixed number or structure on the model. Although each configuration is theoretically plausible, whether these or other patterns emerge remains an empirical question. Accordingly, we pose the following research question:

**Research Question** **1.** *How many latent profiles of university students’ AI attitudes emerge from perceived humanlikeness, adaptability, quality, AI use anxiety, STARA awareness, and AI creative self-efficacy, and what are their defining configurations*?

### 2.3. Personal and Contextual Resources Associated with AI Attitude Profile Membership

AI attitudes develop within a broader system of personal and contextual resources. Social cognitive career theory emphasizes that self-efficacy, learning experiences, goals, and environmental supports jointly shape career interests and actions (Lent et al., 1994). Career construction theory similarly frames adaptive responses as involving readiness, psychosocial resources, and behaviors that help individuals address changing vocational tasks (Savickas, 2013). Contemporary career-guidance scholarship increasingly emphasizes the need to prepare individuals for uncertain and non-linear career environments. Across the life-design, planned happenstance, and career management skills traditions, career development is viewed as an ongoing process that requires adaptation, exploratory learning, and active self-management rather than reliance on fixed career plans (Savickas et al., 2009; Mitchell et al., 1999; Sultana, 2012). From this perspective, students’ responses to AI-related change can be understood not only as technology-related evaluations, but also in relation to the adaptive and self-management resources they can mobilize while preparing for uncertain career futures. We therefore examine four resources that are especially relevant when students confront AI-driven change. Learning agility and digital literacy represent adaptive learning resources, future work self-salience provides future-oriented direction for career preparation, and perceived university digital support represents a contextual resource for exploration and mastery.

*Learning agility.* Learning agility refers to an orientation toward mastering new competencies and adapting one’s learning in response to novel or changing demands (DeRue et al., 2012; Milani et al., 2024). It may help students remain engaged with evolving AI tools, revise ineffective strategies, and interact with AI more effectively.

*Digital literacy.* Digital literacy encompasses the technical, cognitive, and social-emotional competencies required to use digital technologies effectively and appropriately (W. Ng, 2012). In this study, it primarily reflects students’ perceived ability to locate, evaluate, process, and use digital information and tools, which may support more informed and effective engagement with AI.

*Future work self salience.* Future work self salience refers to the clarity and accessibility of an individual’s hoped-for future work identity (Strauss et al., 2012). A clearer future work self may encourage students to monitor AI-related changes, assess their occupational implications, and invest in relevant preparation.

*Perceived University Digital Support.* Perceived university digital support refers to students’ perceptions that their university provides accessible digital resources, guidance, and technical assistance for engaging with unfamiliar technologies. Such support may create opportunities for experimentation and mastery, thereby facilitating more confident engagement with AI.

Collectively, these resources should distinguish profiles characterized by stronger AI capability evaluations and AI creative self-efficacy from profiles marked by weaker evaluations and lower AI creative self-efficacy. Because the exact profile structure cannot be specified a priori, however, more specific profile-to-profile associations, particularly those involving threat-related configurations, remain exploratory.

**Hypothesis** **1.** *Higher learning agility, digital literacy, future work self-salience, and perceived university digital support will be associated with a greater likelihood of membership in AI attitude profiles characterized by stronger capability evaluations and greater AI creative self-efficacy, relative to profiles characterized by weaker capability evaluations and lower AI creative self-efficacy.*

### 2.4. Group Differences in Career Development Outcomes

AI attitude profiles are substantively meaningful when they differ on theoretically relevant career criteria. We examine career crafting and self-perceived employability as complementary indicators of students’ preparation for an AI-affected labor market. Career crafting captures proactive career action, whereas self-perceived employability captures confidence in future labor-market prospects. Together, these variables are consistent with career-development literature emphasizing active career self-management and employability in navigating changing and non-linear career transitions (Savickas et al., 2009; Sultana, 2012).

*Career Crafting.* Career crafting refers to proactive behaviors through which individuals reflect on and shape their career paths, including goal setting, skill development, and networking (Tims & Akkermans, 2020; Lee et al., 2021). Such behaviors support adaptation in changing career contexts (Savickas, 2013; De Vos et al., 2020). Students who perceive AI as capable and who feel more efficacious in using AI may be more inclined to explore AI-related opportunities and develop relevant skills. Perceived threat may have different implications across profiles. When threat appraisals co-occur with lower AI creative self-efficacy, students may be less confident in translating AI-related change into proactive action. In contrast, elevated threat accompanied by stronger AI creative self-efficacy may coexist with more active preparation.

*Self-Perceived Employability.* Self-perceived employability refers to students’ beliefs about their ability to obtain suitable employment based on their capabilities and perceived labor-market conditions (Rothwell et al., 2008). Strong evaluations of AI capability and greater AI creative self-efficacy may be associated with greater confidence in one’s preparedness to work with AI. However, elevated threat need not imply lower employability when it co-occurs with stronger AI creative self-efficacy.

Taken together, profiles characterized by stronger AI capability evaluations and greater AI creative self-efficacy are expected to show more favorable career preparation than profiles characterized by weaker capability evaluations and lower AI creative self-efficacy. However, because perceived threat may either inhibit or motivate preparation depending on the broader configuration, differences among profiles that vary primarily in threat remain exploratory.

**Hypothesis** **2.** *AI attitude profiles characterized by stronger evaluations of AI capability and higher AI creative self-efficacy will report higher levels of career crafting and self-perceived employability than profiles characterized by weaker evaluations of AI capability and lower AI creative self-efficacy.*

### 2.5. The Present Study

Using three-wave data, this study examines how university students’ evaluations of AI capability, perceived threat, and AI creative self-efficacy combine into distinct AI attitude profiles. Learning agility, digital literacy, future work self-salience, and perceived university digital support were assessed as prospective correlates of profile membership. Career crafting and self-perceived employability were assessed to establish the career-development significance of the profiles.

This design extends the AAAW framework from multidimensional measurement to a person-centered account of AI attitudes in the university context. It also integrates cognitive appraisal and vocational adaptation perspectives to examine two theoretically important possibilities: that opportunity and threat may coexist within the same student, and that an absence of perceived threat may reflect either adaptive confidence or disengagement. Because the exact number and form of the profiles cannot be specified a priori, we use an exploratory research question rather than predetermined profile hypotheses.

## 3. Method

### 3.1. Participants and Procedure

Data were collected from April to May 2026 through a three-wave, time-lagged survey of first-year university students in China, with an interval of two weeks between consecutive waves. Participants were recruited by distributing the survey invitation and questionnaire link through WeChat groups for first-year students at the university. A total of 500 eligible students were invited to participate, of whom 468 completed Wave 1, corresponding to an initial response rate of 93.6%. Participation was voluntary, and all questionnaires were administered in Chinese. Students who completed all three waves and whose responses met the prespecified data-quality criteria could scan a QR code on the survey platform to receive an individualized feedback report summarizing their AI- and career-related survey results.

The temporal separation was introduced as a procedural measure intended to reduce some immediate consistency and demand effects across questionnaires (Podsakoff et al., 2003). At Wave 1, participants reported their demographic characteristics, learning agility, digital literacy, future work self-salience, and perceived university digital support. At Wave 2, the six AI attitude profile indicators were assessed. At Wave 3, participants completed the career-development measures.

A total of 468 students completed Wave 1, of whom 432 completed Wave 2 (92.3% of the Wave 1 sample). At Wave 3, 398 responses were received. Across the three waves, 12 cases were excluded during prespecified data-quality screening because of failed attention checks, extended straight-line responding, or implausibly short completion times. After data-quality screening and matching responses across the three waves, 387 participants provided valid matched data, corresponding to a retention rate of 89.6% from Wave 2 and an overall three-wave retention rate of 82.7%. Eight cases exceeded the prespecified multivariate-outlier criterion based on Mahalanobis distance, D^2^ > 22.458 (df = 6, *p* < 0.001), yielding a final analytic sample of 379 participants.

The final sample included 283 women (74.7%) and 96 men (25.3%). All participants were first-year university students enrolled in the broad field of business administration.

### 3.2. Measures

All measures originally developed in English were administered in Chinese following a translation-back-translation procedure conducted by two doctoral students to ensure semantic equivalence. Any discrepancies were discussed and resolved before the survey was finalized. To provide additional evidence of content validity and contextual appropriateness, all items were reviewed by a professor specializing in organizational behavior, a university counselor responsible for student affairs, and five first-year students. The professor assessed the conceptual relevance and alignment of the items with their intended constructs, whereas the counselor and students evaluated contextual suitability, comprehensibility, and wording clarity. Minor revisions were made based on their feedback.

At Wave 2, AI was presented as a broad category of AI-enabled tools and systems that students encounter or use in their learning and everyday life. These included, for example, generative AI tools, conversational AI assistants, and other AI-enabled applications. Participants were not asked to focus on a single platform but to respond with reference to AI technologies with which they were familiar.

*Perceived humanlikeness, adaptability, and quality of AI.* Perceived humanlikeness, adaptability, and quality of AI were measured using three items per dimension adapted and condensed from the AAAW scale developed by Park et al. (2024). The original dimensions were retained because they capture distinct perceptions of AI-specific characteristics, whereas item wording was shortened and contextualized to students’ interactions with AI in learning settings. A sample item of perceived humanlikeness was, “I feel like I’m communicating with another person rather than a program.” A sample item of perceived adaptability was, “The AI tool can continuously improve the quality of its output based on my feedback.” A sample item of perceived quality was, “The responses generated by AI are generally accurate and professional in most cases.” Participants responded on a five-point scale ranging from 1 (strongly disagree) to 5 (strongly agree).

*AI use anxiety.* AI use anxiety was measured using four items developed with reference to Wang and Wang’s (2022) Artificial Intelligence Anxiety Scale. The items assessed students’ anxiety about continually acquiring new AI skills and keeping pace with rapidly changing AI technologies. A sample item was, “I am afraid that I will be unable to keep pace with advances in AI technology.” Participants responded on a five-point scale ranging from 1 (strongly disagree) to 5 (strongly agree).

*STARA awareness.* STARA awareness was measured using four items adapted and contextualized from Brougham and Haar (2018) to reflect university students’ concerns about AI and automation. A sample item was, “I am concerned that the professional skills I am developing may be largely replaced by AI in the near future.” Participants responded on a seven-point scale ranging from 1 (strongly disagree) to 7 (strongly agree).

*AI creative self-efficacy.* AI creative self-efficacy was measured using three items developed with reference to Tierney and Farmer’s (2002) creative self-efficacy scale. The original self-efficacy logic was retained, but the referent was narrowed to students’ confidence in using AI to generate creative academic outcomes. A sample item was, “I am confident that I can use AI tools to produce more innovative academic work.” Participants responded on a seven-point scale ranging from 1 (strongly disagree) to 7 (strongly agree).

*Learning agility.* Learning agility was assessed using six items developed for the present study with reference to Milani et al.’s (2024) Learning Agility at Work Scale. The original scale was designed for employees and emphasizes adaptability, openness to learning, and adjustment in response to experience. We retained this core conceptual content while rewriting the items to refer to learning strategies and academic challenges relevant to first-year university students. A sample item was, “When a learning method or strategy does not work, I can quickly adjust and try a new approach.” Participants responded on a five-point scale ranging from 1 (strongly disagree) to 5 (strongly agree).

*Digital literacy.* Digital literacy was assessed using four items adapted and contextualized from W. Ng’s (2012) digital literacy framework. A sample item was, “I can use search engines or AI tools to efficiently screen and analyze information.” Participants responded on a five-point scale ranging from 1 (strongly disagree) to 5 (strongly agree).

*Future work self salience.* Future work self-salience was measured with five items adapted from Strauss et al. (2012). A sample item was, “I can easily imagine my future work self.” Participants responded on a five-point scale ranging from 1 (strongly disagree) to 5 (strongly agree).

*Perceived university digital support.* Perceived university digital support was measured with three items developed with reference to organizational support theory (Eisenberger et al., 1986). The construct was adapted to the university context by treating the institution as a source of instrumental and developmental support for students’ engagement with unfamiliar digital technologies. A sample item was, “When I encounter difficulties while exploring unfamiliar digital domains, my university provides access to mentoring or platform support.” Participants responded on a five-point scale ranging from 1 (strongly disagree) to 5 (strongly agree).

*Career crafting.* Career crafting was measured using 20 items adapted from Lee et al.’s (2021) Career Crafting Assessment and contextualized for first-year students beginning to explore and prepare for future careers. The measure retained four domains: proactive career development, relational resource mobilization, positive career-meaning reflection, and adaptive career adjustment. Sample items included “I actively develop new skills that are relevant to my career goals” and “I adjust my career plans when facing changes or obstacles.” Participants responded on a five-point scale ranging from 1 (strongly disagree) to 5 (strongly agree).

*Self-perceived employability.* Self-perceived employability was measured using three items adapted from Rothwell et al. (2008). The items focused on students’ confidence in obtaining desirable employment and in possessing skills valued by employers. A sample item was, “I believe that I possess the skills and abilities employers are looking for.” Participants responded on a five-point scale ranging from 1 (strongly disagree) to 5 (strongly agree).

Table 1 summarizes the key reliability and validity statistics for all measures, including Cronbach’s alpha, standardized factor loadings, composite reliability (CR), and average variance extracted (AVE). Overall, the measures demonstrated satisfactory internal consistency, with CR values ranging from 0.72 to 0.96 and Cronbach’s α coefficients ranging from 0.69 to 0.96. Standardized factor loadings were generally acceptable, and AVE values met or exceeded the conventional 0.50 criterion for all constructs except perceived humanlikeness (AVE = 0.48), whose CR nevertheless remained acceptable (0.72). The results provide generally adequate evidence of reliability and convergent validity for the study measures.

### 3.3. Analytical Strategy

All analyses were conducted in Mplus 9.0 using the final analytic sample of 379 participants. Descriptive statistics, zero-order correlations, and Cronbach’s α coefficients were first calculated.

Before conducting LPA, we examined the distinctiveness of the six profile constructs using item-level confirmatory factor analysis (CFA) with robust maximum likelihood (MLR) estimation. Model fit was evaluated using the robust χ^2^, CFI, TLI, RMSEA with its 90% confidence interval, SRMR, AIC, BIC, and sample-size-adjusted BIC (Hu & Bentler, 1999).

For LPA, the six profile indicators were standardized so that profile shape could be interpreted relative to the sample mean. To determine the best-fitting number of profiles, we constructed various profile solutions ranging from one to eight profiles (Nylund et al., 2007) with MLR. To reduce the risk of local maxima, 7000 random sets of starting values were generated, and the 200 best solutions were retained for final-stage optimization (Hipp & Bauer, 2006). All reported solutions converged and replicated the best log likelihood. In all latent profile models, indicator means were freely estimated across profiles, indicator variances were constrained to equality across profiles, and within-profile covariances were fixed to zero, corresponding to a diagonal, profile-invariant covariance structure.

For each profile solution, we examined the fit information criteria. Following established recommendations for LPA (Nylund et al., 2007; Spurk et al., 2020), statistical indices included the Akaike information criterion (AIC; Akaike, 1987), Bayesian information criterion (BIC; Schwarz, 1978), sample-size-adjusted BIC (SABIC; Sclove, 1987), adjusted Lo-Mendell-Rubin likelihood ratio test (LMR; Lo et al., 2001), and bootstrap likelihood ratio test (BLRT; McLachlan & Peel, 2000). Lower AIC, BIC, and SABIC values indicated better relative fit. Significant LMR and BLRT results indicated that a k-profile solution fit better than the corresponding k-1-profile solution, whereas a nonsignificant result favored retention of the k-1-profile solution. Entropy and average posterior probabilities were used to evaluate classification precision (Entropy ≥ 0.60; Average Posterior Probability of Assignment (APPA) ≥ 0.70). Profile size, parsimony, and substantive interpretability were also considered.

After selecting the final solution, prospective correlates of profile membership were analyzed with the automatic R3STEP procedure, which estimates multinomial logistic regressions while accounting for profile-classification error (Asparouhov & Muthén, 2014; Vermunt, 2010). Career-development correlates were compared across profiles using the Bolck-Croon-Hagenaars (BCH) auxiliary function and Wald χ^2^ tests, which preserve the measurement model while adjusting for classification uncertainty (Asparouhov & Muthén, 2014; Bakk et al., 2013).

## 4. Results

### 4.1. Preliminary Analyses

Table 2 presents descriptive statistics and zero-order correlations. The sample size (N = 379) was considered adequate for LPA, as prior recommendations suggest that approximately 300 to 500 participants are generally sufficient for such analyses (Ferguson et al., 2020). The three AI capability evaluations were moderately to strongly intercorrelated (r_s_ = 0.56–0.68), and were positively associated with AI creative self-efficacy (r_s_ = 0.40–0.44). AI use anxiety and STARA awareness were also strongly correlated (r = 0.60), but each showed only weak positive associations with the capability evaluations (r_s_ = 0.13–0.22) and no significant associations with AI creative self-efficacy. This pattern provided preliminary support for distinguishing capability evaluations, perceived threat, and AI creative self-efficacy.

Before profile estimation, an item-level CFA was conducted to evaluate the measurement structure of the six profile indicators. The hypothesized correlated six-factor model fit the data well, robust χ^2^(155) = 207.84, CFI = 0.982, TLI = 0.978, RMSEA = 0.030, 90% CI [0.018, 0.040], and SRMR = 0.035. These findings supported the six-factor structure and the use of the six constructs as separate indicators in the subsequent LPA.

### 4.2. Latent Profile Analysis of AI Attitudes

Table 3 presents the fit statistics for the one- through eight-profile solutions. AIC and SABIC decreased with additional profiles, whereas BIC reached its lowest value for the seven-profile solution. Thus, the information criteria did not indicate a single unambiguous solution. The adjusted LMR test remained significant through the four-profile solution, *p* = 0.035, but became nonsignificant for the five-profile solution, *p* = 0.376, and remained nonsignificant thereafter. This pattern indicated that adding a fifth or subsequent profile did not significantly improve fit over the preceding solution.

The BLRT was significant for all multi-profile solutions and therefore did not provide a clear stopping point. The four-profile solution showed adequate classification precision, with an entropy value of 0.824 and average posterior probabilities ranging from 0.883 to 0.949. Its smallest profile represented 6.1% of the sample. More complex solutions produced progressively smaller profiles without yielding substantively distinct configurations. On balance, the four-profile solution was retained based on statistical evidence, profile size, parsimony, and interpretability. All candidate models converged, and the best log likelihood was successfully replicated.

### 4.3. Description of the Four AI Attitude Profiles

**Figure 1:**
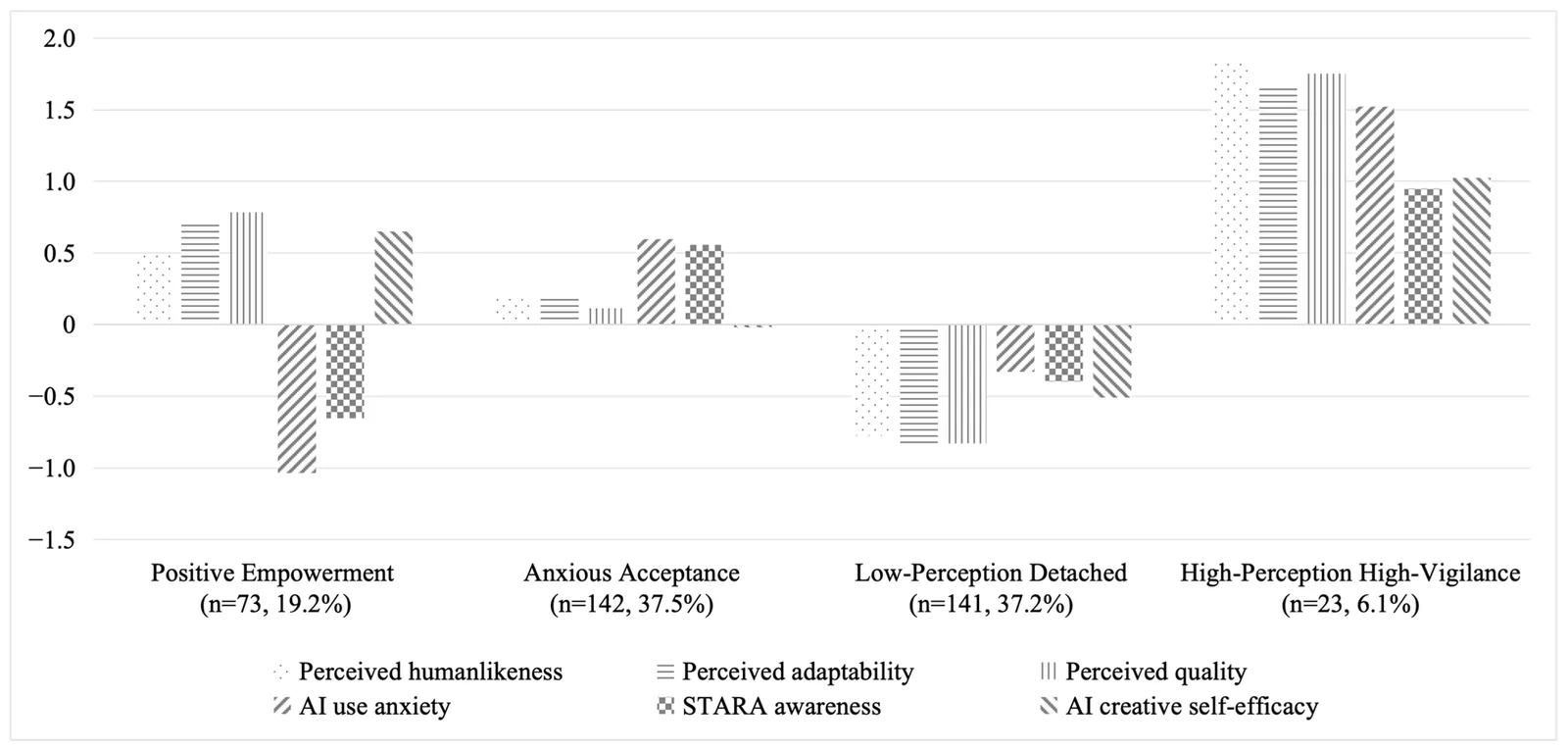
Latent Profiles of Overall Attitudes Toward Artificial Intelligence. Note. Standardized means for the six indicators across the four-profile solution.

Standardized indicator means are presented in Table 4 and Figure 1. Profile names reflect the relative configuration of the six indicators.

*“Positive Empowerment (n = 73, 19.2%).”* This profile was characterized by elevated AI creative self-efficacy and favorable capability perceptions, especially adaptability and quality. In contrast, AI use anxiety and STARA awareness were substantially below the sample mean and were the lowest across the four profiles. This pattern represents an opportunity-oriented configuration in which high AI creative self-efficacy and capability evaluations coexist with low perceived threat.

*“Anxious Acceptance (n = 142, 37.5%).”* Students in this profile reported approximately average to slightly above-average evaluations of AI capability and average AI creative self-efficacy. However, both AI use anxiety and STARA awareness were moderately above the sample mean. The profile is therefore distinguished less by strongly favorable evaluations than by the coexistence of modest acceptance and elevated threat. It reflects students who recognize some AI capability while remaining concerned about using AI and its broader occupational implications.

*“Low-Perception Detached (n = 141, 37.2%).”* This profile showed consistently below-average scores across all six indicators. The largest deviations occurred for perceived humanlikeness, adaptability, and quality, while AI creative self-efficacy was also below average. AI use anxiety and STARA awareness were relatively low, but not as low as in the Positive Empowerment profile. Thus, the low perceived threat characterizing this profile occurred alongside weak capability evaluations and limited confidence in using AI, suggesting low engagement with AI-related change.

*“High-Perception High-Vigilance (n = 23, 6.1%).”* This profile showed markedly above-average scores on all six indicators. Perceived humanlikeness, adaptability, and quality were especially high, indicating strong perceptions of AI capability, while AI creative self-efficacy was also substantially above average. The latter provides the clearest positive, opportunity-oriented component of the profile, reflecting students’ confidence in their capability to use AI creatively. At the same time, these students reported the highest levels of AI use anxiety and STARA awareness. Thus, this profile combines a positive, efficacy-based orientation toward AI use, supported by strong perceptions of AI capability, with pronounced use-related anxiety and career-related threat awareness. This pattern provides suggestive configurational evidence of opportunity-threat coactivation, showing that confidence in using AI creatively can coexist with heightened vigilance regarding its demands and career implications. Given the relatively small size of this profile (*n* = 23, 6.1%), however, its characteristics should be interpreted cautiously pending replication in larger samples.

Taken together, the four profiles differed in both level and shape. *High-Perception High-Vigilance* and *Low-Perception Detached* showed broadly elevated and reduced scores, respectively, whereas *Positive Empowerment* and *Anxious Acceptance* reflected more differentiated combinations of capability evaluations, threat, and AI creative self-efficacy. Notably, favorable capability evaluations and high AI creative self-efficacy coexisted with either low or high threat, while low threat characterized both empowered and detached configurations.

### 4.4. Prospective Correlates of Latent Profile Membership

Table 5 presents the R3STEP results for the four variables. The odds ratios represent the change in relative odds of profile membership associated with a one-standard-deviation increase in each variable.

Learning agility showed the clearest differentiation across profiles. Higher learning agility was associated with greater odds of *Positive Empowerment* relative to *Anxious Acceptance* (OR = 1.899) and *Low-Perception Detached* (OR = 4.873). It was also associated with greater odds of *Anxious Acceptance* relative to *Low-Perception Detached* (OR = 2.566) and lower odds of *Anxious Acceptance* (OR = 0.315) and *Low-Perception Detached* (OR = 0.123) relative to *High-Perception High-Vigilance*.

Higher digital literacy was associated with greater odds of *Positive Empowerment* rather than *Anxious Acceptance* (OR = 1.444) or *Low-Perception Detached* (OR = 1.779). It was also associated with lower odds of *Anxious Acceptance* (OR = 0.421) and *Low-Perception Detached* (OR = 0.342) relative to *High-Perception High-Vigilance*. Overall, it primarily differentiated the two profiles with stronger capability evaluations and AI creative self-efficacy from the remaining profiles.

Higher future work self-salience was associated with lower odds of other profiles (OR_s_ = 0.370, 0.372, 0.208) relative to *High-Perception High-Vigilance*. It was also associated with greater odds of *Positive Empowerment* (OR = 1.783) and *Anxious Acceptance* (OR = 1.791) relative to *Low-Perception Detached*.

Perceived university digital support primarily distinguished *Low-Perception Detached* from the other profiles. Higher support was associated with greater odds of *Positive Empowerment* relative to *Anxious Acceptance* (OR = 1.817) and *Low-Perception Detached* (OR = 3.728), and of *Anxious Acceptance* relative to *Low-Perception Detached* (OR = 2.052). It was also associated with lower odds of *Low-Perception Detached* relative to *High-Perception High-Vigilance* (OR = 0.247).

Overall, ***Hypothesis 1*** received substantial support. Higher levels of each variable were associated with greater odds of membership in *Positive Empowerment* and *High-Perception High-Vigilance* rather than *Low-Perception Detached*. Learning agility, future work self-salience, and perceived university digital support also favored *Anxious Acceptance* over *Low-Perception Detached*, whereas digital literacy did not significantly differentiate these two profiles. Thus, higher levels of the prospective correlates were generally associated with profiles characterized by stronger capability evaluations and greater AI creative self-efficacy. However, they were not consistently associated with lower perceived threat, as *High-Perception High-Vigilance* combined elevated AI use anxiety and STARA awareness with stronger personal and contextual resources.

### 4.5. Career-Development Differences Across Latent Profiles

**Figure 2:**
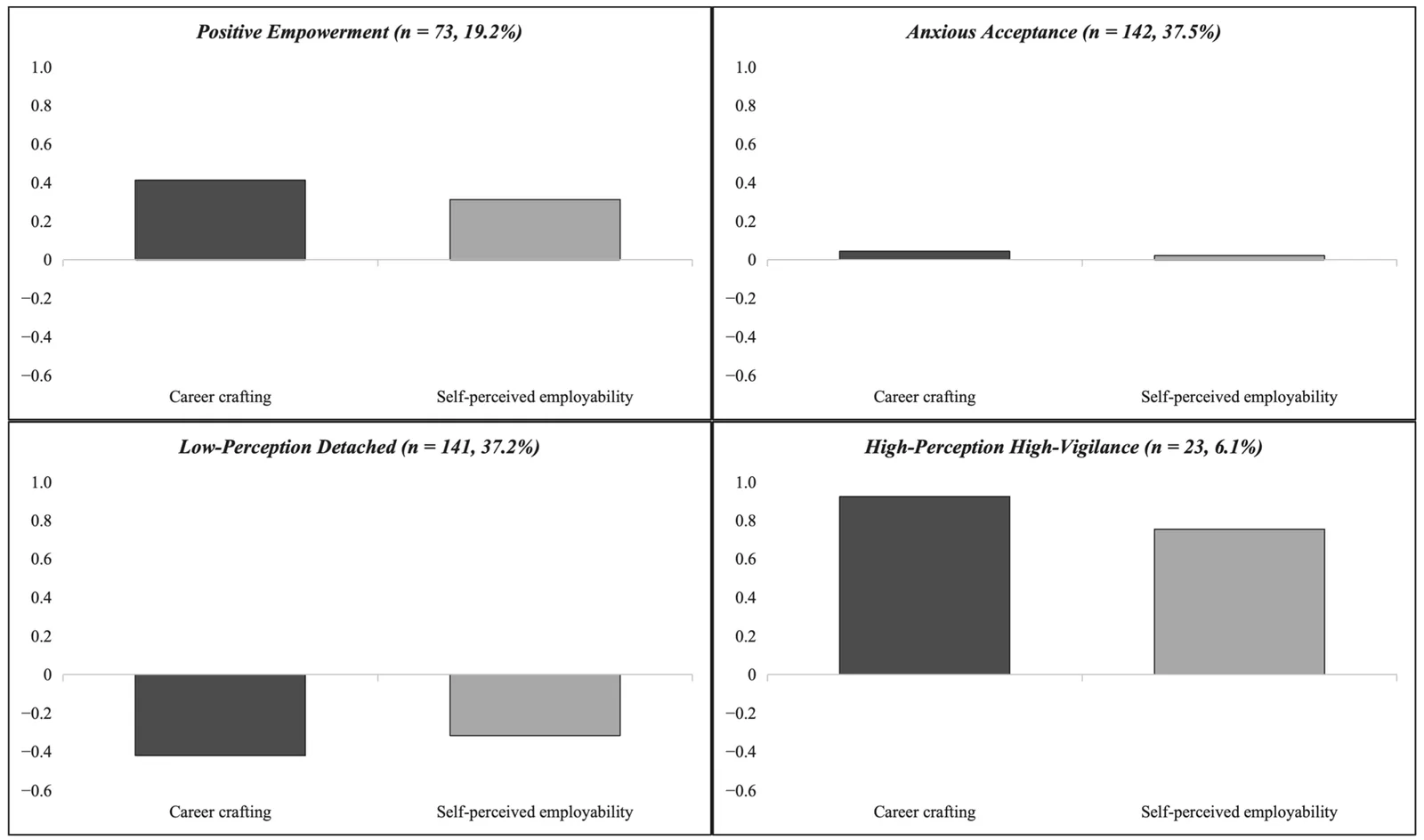
Standardized Career-Development Correlates Across the Four AI-Attitude Profiles. Note. Values are standardized profile-specific means for career crafting and self-perceived employability.

Table 6 presents the BCH-adjusted means for career crafting and self-perceived employability. The omnibus Wald tests indicated significant profile differences in career crafting, χ^2^(3) = 52.841, *p* < 0.001, and self-perceived employability, χ^2^(3) = 27.734, *p* < 0.001. Figure 2 illustrates the corresponding standardized profile-specific means.

For career crafting, *High-Perception High-Vigilance* (M = 4.22) and *Positive Empowerment* (M = 3.91) did not differ significantly, but both reported higher levels than *Anxious Acceptance* (M = 3.69) and *Low-Perception Detached* (M = 3.41). *Anxious Acceptance* also exceeded *Low-Perception Detached*. Thus, the two profiles characterized by stronger capability evaluations and AI creative self-efficacy showed the highest levels of proactive career behavior, despite differing substantially in perceived threat.

For self-perceived employability, *High-Perception High-Vigilance* (M = 3.96) reported higher levels than *Anxious Acceptance* (M = 3.40) and *Low-Perception Detached* (M = 3.14), but did not differ significantly from *Positive Empowerment* (M = 3.62). *Positive Empowerment* and *Anxious Acceptance* also exceeded *Low-Perception Detached*, although they did not differ significantly from each other.

Overall, ***Hypothesis 2*** was supported. Profiles characterized by stronger capability evaluations and greater AI creative self-efficacy generally showed more favorable career-development correlates than *Low-Perception Detached*. Notably, *Positive Empowerment* and *High-Perception High-Vigilance* did not differ significantly on either outcome, despite their contrasting threat levels. This pattern suggests that elevated AI-related threat is not necessarily incompatible with career preparation when it co-occurs with favorable capability evaluations and high AI creative self-efficacy. Conversely, the relatively low threat reported by *Low-Perception Detached* was not associated with higher levels of career crafting or employability beliefs.

## 5. Discussion

Using three-wave data, this study examined how first-year business-administration students’ evaluations of AI capability, perceived threat, and AI creative self-efficacy combine into distinct AI attitude profiles. Four profiles emerged. *Positive Empowerment* combined favorable capability evaluations and high AI creative self-efficacy with low threat. *Anxious Acceptance* combined moderately favorable evaluations with elevated AI use anxiety and STARA awareness. *Low-Perception Detached* showed broadly low levels across capability evaluations, threat, and AI creative self-efficacy. *High-Perception High-Vigilance* was elevated across all six indicators, combining strong capability evaluations and AI creative self-efficacy with pronounced threat.

The four prospective correlates systematically differentiated profile membership, particularly separating *Low-Perception Detached* from the more engaged profiles. The profiles also differed in subsequent career crafting and self-perceived employability. *Positive Empowerment* and *High-Perception High-Vigilance* showed similarly favorable levels of both career criteria, whereas *Low-Perception Detached* reported the lowest levels. Within the present sample, these findings support a configurational rather than purely positive-to-negative understanding of AI attitudes.

### 5.1. Theoretical Implications

First, this study advances a configurational and bivalent understanding of AI attitudes. Existing research conceptualizes attitudes toward workplace AI as multidimensional, involving capability evaluations, affective reactions, perceived threat, and personal utility (Park et al., 2024). Our findings extend this perspective by showing that, within the present sample, AI capability perceptions, threat-related responses, and AI creative self-efficacy form qualitatively distinct within-person configurations rather than positions on a single positive-negative continuum. Most notably, the *High-Perception High-Vigilance* profile combined very high AI creative self-efficacy with strong perceptions of AI capability and pronounced AI use anxiety and replacement concerns. The *Anxious Acceptance* profile likewise combined moderately favorable evaluations with elevated threat. Consistent with the evaluative-space model, positive and negative evaluations can coexist because they are partly independent rather than mutually exclusive (Cacioppo & Berntson, 1994). Thus, enthusiasm and concern should not be interpreted as contradictory responses. A person-centered approach reveals forms of evaluative complexity that would be obscured by an overall AI attitude score.

Second, the findings distinguish low perceived threat from genuine adaptive readiness. Although the *Low-Perception Detached* profile reported relatively little AI use anxiety and STARA awareness, it also showed the lowest AI creative self-efficacy, career crafting, and perceived employability. Appraisal and protection motivation perspectives distinguish threat appraisal from coping appraisal: adaptive responses depend not only on whether a change is perceived as threatening, but also on whether individuals regard it as personally relevant and believe they possess sufficient coping resources (Lazarus & Folkman, 1984; Maddux & Rogers, 1983). Low anxiety may therefore reflect low salience or disengagement rather than successful adaptation. AI fatigue offers a complementary explanation for such disengagement. Lau et al. (2026) conceptualize behavioral avoidance and disengagement as manifestations of AI fatigue and found greater fatigue to be associated with lower current AI use and stronger intentions to reduce use. Thus, for some students, apparent detachment may also reflect fatigue-related withdrawal. This finding challenges the assumption that the absence of threat is inherently positive and suggests that AI readiness should be defined by the joint presence of awareness, efficacy, and active career engagement.

Third, the profiles reveal two contrasting configurations associated with proactive career adaptation. *Positive Empowerment* represents a resource-dominant configuration, characterized by favorable AI evaluations, strong efficacy, low threat, and positive career correlates. In contrast, *High-Perception High-Vigilance* represents a high-vigilance configuration: students reported similarly high career crafting and employability while also experiencing substantially greater anxiety. This pattern is broadly consistent with proactive motivation theory, which proposes that proactive action may be supported by both “can do” states, such as perceived capability, and activated “energized to” states that facilitate proactive goal pursuit (Parker et al., 2010). Likewise, job demands-resources theory indicates that personal resources may sustain proactive behavior without fully offsetting the psychological costs of high demands (Bakker & Demerouti, 2017). The findings therefore distinguish behavioral effectiveness from psychological sustainability. AI fatigue provides a more specific lens on this sustainability concern. Lau et al. (2026) conceptualize AI fatigue as a psychophysiological state that can emerge when sustained interaction with AI imposes accumulating cognitive and emotional demands. Accordingly, if the strong engagement and vigilance characterizing *High-Perception High-Vigilance* persist, they may carry a longer-term risk of fatigue despite students’ high AI creative self-efficacy. Future longitudinal research should examine whether this profile is transitional or persistent and whether sustained vigilance is associated with subsequent fatigue and changes in career adaptation. Given the small number of students in the *High-Perception High-Vigilance* profile, this interpretation should be regarded as provisional. Replication in larger samples is needed to determine whether this configuration represents a stable and reproducible subgroup.

Fourth, the prospective associations position AI attitude profiles within broader career-development processes. Learning agility and digital literacy can be understood as capacities for continued learning and adjustment, future work self salience as a future-oriented resource for career preparation, and institutional support as a contextual resource for exploration and mastery. This pattern is consistent with social cognitive career theory, which emphasizes the role of personal and contextual resources in career development (Lent et al., 1994). More broadly, career-development scholarship highlights adaptation, exploratory learning, and active self-management as important for navigating uncertain and non-linear career environments (Savickas et al., 2009; Mitchell et al., 1999; Sultana, 2012). It also aligns with research showing that salient future work selves are associated with proactive career behavior (Strauss et al., 2012) and that AI literacy involves not only technical knowledge but also critical understanding and effective application (D. T. K. Ng et al., 2021). Future work self-salience was particularly strongly associated with membership in the *High-Perception High-Vigilance*, suggesting that students with a more salient future career identity may also be more attuned to both AI opportunities and threats. Institutional support, by contrast, differentiated *Positive Empowerment* from *Anxious Acceptance*, implying that more supportive educational environments were associated with a more efficacy-oriented and less anxiety-dominant AI attitude configuration. Taken together, these associations suggest that students’ AI attitudes may be embedded in broader processes of career preparation and adaptation to an uncertain future of work, rather than functioning solely as technology-specific evaluations.

### 5.2. Practical Implications

The four profiles suggest that universities may consider replacing uniform AI-literacy programs with differentiated support. For *Low-Perception Detached* students, the priority is to increase the salience and career relevance of AI through discipline-specific tasks, guided reflection, and mastery experiences that strengthen AI creative self-efficacy. *Anxious Acceptance* students already recognize AI’s value but need structured skill development, realistic labor-market information, and strategies for managing uncertainty. *Positive Empowerment* students appear comparatively ready, but should still be encouraged to maintain critical evaluation rather than equating confidence with uncritical trust. For *High-Perception High-Vigilance* students, support should focus on psychological sustainability by helping them set bounded learning goals, manage information overload, and distinguish controllable preparation from broader labor-market uncertainty.

At the institutional level, AI education should be integrated with career development rather than treated as a separate technical service. Academic programs and career centers could jointly provide discipline-specific AI simulations, workshops on augmentation and substitution scenarios, and career-planning activities linked to students’ future work selves. Counseling and well-being services should also be incorporated where anxiety becomes persistent or impairs functioning. Institutional communication should remain balanced: overly optimistic messages may encourage uncritical reliance, whereas alarmist messages may intensify anxiety without improving preparedness.

Finally, the goal of intervention should not be to make all students uniformly positive about AI. Effective support should promote accurate appraisal, strong self-efficacy, proactive career behavior, and psychological sustainability. Accordingly, program evaluation should assess not only AI literacy and confidence, but also career crafting, perceived employability, anxiety, and critical use. Because students may move between profiles as their skills and career goals develop, profile-based support should remain flexible, and profile labels should not be treated as fixed categories.

In particular, recommendations concerning the relatively small *High-Perception High-Vigilance* subgroup should be viewed as provisional. More broadly, the practical implications are best regarded as considerations for comparable first-year student populations, with their wider applicability requiring replication in larger and more diverse samples.

### 5.3. Limitations and Future Research

Several limitations should be considered. First, the sample was relatively homogeneous, comprising first-year business-administration students from one university in China, with women constituting the majority. The identified profile structure, profile prevalence, and associated variables may therefore be partly context-dependent. Future research should examine the replicability of these findings across different disciplines, year levels, institutions, cultural settings, and more gender-balanced samples.

Second, although the three-wave design temporally separated the measurement occasions, it did not establish directionality or causality. Reliance on self-report data and the short two-week intervals do not eliminate common-method variance. Thus, the Wave 1 variables and Wave 3 career measures should be interpreted as prospective and time-lagged correlates, respectively. Moreover, because the AI attitude indicators were assessed at only one wave, profile stability and change could not be examined. Future research should use repeated, longer-term, and multi-source assessments to examine profile stability, change, and longer-term career implications.

Third, the *High-Perception High-Vigilance* profile contained only 23 students (6.1% of the sample). Although the overall classification quality was satisfactory, the small size of this subgroup warrants caution in interpreting its characteristics and associated outcomes. Replication in larger and more diverse samples, together with formal tests of cross-sample profile similarity, is needed to determine whether this configuration can be reliably reproduced.

Fourth, the present study used abbreviated and context-adapted indicators rather than the full AAAW scale of Park et al. (2024). STARA awareness was used as a contextually relevant indicator of career-related threat, whereas AI creative self-efficacy was included as an adjacent personal-capability belief. Neither should be regarded as a direct equivalent of the job-insecurity or personal-utility dimensions of the original AAAW framework. These adaptations may limit direct comparability with studies using the original AAAW scale. Future research should examine whether similar profiles emerge using the full AAAW dimensions or alternative student-focused measures.

Fifth, career crafting and self-perceived employability capture important but subjective aspects of career preparation. Future studies should include behavioral and objective criteria, such as demonstrated AI competence, actual AI use, academic performance, internship attainment, and employment transitions. Measures of psychological well-being would also help determine whether *High-Perception High-Vigilance* reflects productive vigilance, sustained strain, or a temporary stage of adaptation.

## 6. Conclusions

University students’ AI attitudes cannot be reduced to simply liking or disliking AI. Instead, students combine evaluations of AI capability, perceived threat, and AI creative self-efficacy in distinct ways. The four profiles showed that favorable capability evaluations can coexist with either low or high threat, while low threat may reflect either empowered confidence or relative detachment. Personal and contextual resources were prospectively associated with membership in these profiles, and the profiles showed distinct time-lagged associations with career crafting and perceived employability. A configurational perspective therefore offers a more precise foundation for understanding and supporting career development in an AI-shaped labor market.

## Figures and Tables

**Table 1:** Reliability and Validity Statistics for Measures.

Construct	Cronbach’s α	Loading Range	CR	AVE
Perceived humanlikeness	0.69	0.482–0.920	0.72	0.48
Perceived adaptability	0.83	0.740–0.827	0.83	0.63
Perceived quality	0.76	0.680–0.789	0.76	0.52
AI use anxiety	0.91	0.731–0.911	0.91	0.71
STARA awareness	0.89	0.737–0.885	0.89	0.67
AI creative self-efficacy	0.84	0.755–0.848	0.84	0.63
Learning agility	0.90	0.688–0.837	0.90	0.60
Digital literacy	0.80	0.547–0.800	0.81	0.52
Future work self salience	0.90	0.632–0.892	0.90	0.66
Perceived university digital support	0.87	0.816–0.844	0.87	0.69
Career crafting	0.96	0.684–0.813	0.96	0.56
Self-perceived employability	0.87	0.806–0.858	0.87	0.70

**Table 2:** Descriptive Statistics and Correlations Among Study Variables.

Variable	M	SD	1	2	3	4	5	6	7	8	9	10	11	12
1. Perceived humanlikeness	3.43	0.72	-	-	-	-	-	-	-	-	-	-	-	-
2. Perceived adaptability	3.66	0.72	0.61 ***	-	-	-	-	-	-	-	-	-	-	-
3. Perceived quality	3.55	0.69	0.56 ***	0.68 ***	-	-	-	-	-	-	-	-	-	-
4. AI use anxiety	3.08	0.92	0.22 ***	0.15 **	0.13 *	-	-	-	-	-	-	-	-	-
5. STARA awareness	4.79	1.17	0.20 ***	0.16 **	0.16 **	0.60 ***	-	-	-	-	-	-	-	-
6. AI creative self-efficacy	5.06	1.03	0.43 ***	0.40 ***	0.44 ***	−0.08	0.05	-	-	-	-	-	-	-
7. Learning agility	3.69	0.64	0.38 ***	0.42 ***	0.46 ***	−0.02	0.07	0.58 ***	-	-	-	-	-	-
8. Digital literacy	3.91	0.67	0.22 ***	0.22 ***	0.21 ***	−0.05	0.11 *	0.46 ***	0.48 ***	-	-	-	-	-
9. Future work self salience	3.22	0.81	0.33 ***	0.31 ***	0.24 ***	0.12 *	0.09	0.43 ***	0.52 ***	0.33 ***	-	-	-	-
10. Perceived university digital support	3.75	0.75	0.31 ***	0.33 ***	0.34 ***	−0.01	0.09	0.41 ***	0.54 ***	0.35 ***	0.36 ***	-	-	-
11. Career crafting	3.66	0.61	0.28 ***	0.28 ***	0.29 ***	−0.02	0.02	0.46 ***	0.57 ***	0.44 ***	0.43 ***	0.39 ***	-	-
12. Self-perceived employability	3.38	0.77	0.24 ***	0.27 ***	0.24 ***	−0.02	−0.03	0.42 ***	0.45 ***	0.35 ***	0.45 ***	0.30 ***	0.63 ***	-

*Note.* N = 379. Values below the diagonal are zero-order correlations. * *p* < 0.05. ** *p* < 0.001. *** *p* < 0.001.

**Table 3:** Fit Indices for the Latent Profile Models.

Profiles	LL	FP	AIC	BIC	SaBIC	Entropy	LMR (*p*)	BLRT (*p*)	Smallest Profile, *n* (%)
1	−3223.662	12	6471.325	6518.575	6480.501				379 (100.0%)
2	−3051.010	19	6140.020	6214.833	6154.550	0.725	<0.001	<0.001	188 (49.6%)
3	−2956.551	26	5965.101	6067.477	5984.985	0.841	<0.001	<0.001	30 (7.9%)
4	−2896.637	33	5859.275	5989.213	5884.511	0.824	0.035	<0.001	23 (6.1%)
5	−2863.548	40	5807.095	5964.597	5837.685	0.840	0.376	<0.001	18 (4.7%)
6	−2840.742	47	5775.484	5960.548	5811.427	0.842	0.602	<0.001	16 (4.2%)
7	−2819.259	54	5746.519	5959.146	5787.815	0.815	0.298	<0.001	12 (3.2%)
8	−2805.991	61	5733.982	5974.171	5780.631	0.825	0.407	0.018	5 (1.3%)

*Note.* N = 379. LL = log likelihood; FP = free parameters; AIC = Akaike information criterion; BIC = Bayesian information criterion; SaBIC = sample-size-adjusted BIC; LMR (*p*) = *p*-value for the adjusted Lo-Mendell-Rubin-test; BLRT (*p*) = *p*-value for the bootstrapped likelihood ratio test.

**Table 4:** Standardized Indicator Means for the Four AI Attitude Profiles.

Profile	Perceived Humanlikeness	Perceived Adaptability	Perceived Quality	AI Use Anxiety	STARA Awareness	AI Creative Self-Efficacy
Positive Empowerment (*n* = 73, 19.2%)	0.495	0.704	0.783	−1.037	−0.653	0.651
Anxious Acceptance (*n* = 142, 37.5%)	0.198	0.190	0.112	0.596	0.557	−0.008
Low-Perception Detached (*n* = 141, 37.2%)	−0.778	−0.857	−0.831	−0.328	−0.398	−0.511
High-Perception High-Vigilance (*n* = 23, 6.1%)	1.822	1.674	1.753	1.521	0.951	1.025

*Note.* Values are standardized means relative to the final analytic sample. Positive values indicate scores above the sample mean, and negative values indicate scores below the sample mean.

**Table 5:** Results of R3STEP Models Examining Associations with Profile Membership.

Prospective Correlates	A vs. B b (SE)	OR	A vs. C b (SE)	OR	A vs. D b (SE)	OR	B vs. C b (SE)	OR	B vs. D b (SE)	OR	C vs. D b (SE)	OR
Learning agility	0.641 ** (0.208)	1.899	1.584 *** (0.246)	4.873	−0.514 (0.439)	0.598	0.942 *** (0.179)	2.566	−1.156 ** (0.445)	0.315	−2.098 *** (0.485)	0.123
Digital literacy	0.368 * (0.160)	1.444	0.576 ** (0.168)	1.779	−0.497 (0.344)	0.609	0.208 (0.150)	1.231	−0.864 * (0.334)	0.421	−1.072 ** (0.341)	0.342
Future work self salience	−0.005 (0.200)	0.995	0.578 ** (0.191)	1.783	−0.993 * (0.393)	0.370	0.583 *** (0.163)	1.791	−0.988 ** (0.353)	0.372	−1.571 *** (0.371)	0.208
Perceived university digital support	0.597 ** (0.191)	1.817	1.316 *** (0.234)	3.728	−0.084 (0.475)	0.919	0.719 *** (0.189)	2.052	−0.681 (0.473)	0.506	−1.400 ** (0.514)	0.247

*Note.* N = 379. A = Positive Empowerment; B = Anxious Acceptance; C = Low-Perception Detached; D = High-Perception High-Vigilance. b = multinomial logit coefficient; SE = standard error; OR = odds ratio for membership in the first-listed profile relative to the second-listed profile. * *p* < 0.05. ** *p* < 0.01. *** *p* < 0.001.

**Table 6:** BCH-Adjusted Career-Development Correlates Across the Four Profiles.

Outcome	Positive Empowerment (A)	Anxious Acceptance (B)	Low-Perception Detached (C)	High-Perception High-Vigilance (D)	Overall Wald χ^2^
Career crafting	3.912 (0.077) ^BC^	3.689 (0.057) ^ACD^	3.407 (0.045) ^ABD^	4.222 (0.159) ^BC^	52.841 ***
Self-perceived employability	3.624 (0.094) ^C^	3.400 (0.070) ^CD^	3.140 (0.066) ^ABD^	3.964 (0.208) ^BC^	27.734 ***

*Note.* N = 379. Values are BCH-adjusted means with standard errors in parentheses. Overall tests have df = 3. Superscript profile letters indicate significant pairwise differences at *p* < 0.05. *** *p* < 0.001.

## Data Availability

The data presented in this study are available on request from the corresponding author due to privacy and confidentiality restrictions.
